# Phospholipase C delta 1 inhibits WNT/β‐catenin and EGFR-FAK-ERK signaling and is disrupted by promoter CpG methylation in renal cell carcinoma

**DOI:** 10.1186/s13148-023-01448-2

**Published:** 2023-02-27

**Authors:** Jianlian Xie, Jun Zhou, Jiliang Xia, Ying Zeng, Guo Huang, Weihong Zeng, Tingyu Fan, Lili Li, Xi Zeng, Qian Tao

**Affiliations:** 1 Cancer Epigenetics Laboratory, Department of Clinical Oncology, State Key Laboratory of Translational Oncology, Sir YK Pao Center for Cancer and Li Ka Shing Institute of Health Sciences, The Chinese University of Hong Kong, Hong Kong, China; 2 Hunan Province Key Laboratory of Tumor Cellular and Molecular Pathology, Cancer Research Institute, Hengyang Medical School, University of South China, Hengyang, 421001 Hunan China; 3 Department of Burn and Plastic Surgery, General Hospital of Southern Theater Command, PLA, Guangzhou, China

**Keywords:** *PLCD1*, CpG methylation, WNT/β-catenin, EMT, RCC

## Abstract

**Background:**

*PLCD1*, located at 3p22, encodes an enzyme that mediates cellular metabolism and homeostasis, intracellular signal transduction and movement. *PLCD1* plays a pivotal role in tumor suppression of several types of cancers; however, its expression and underlying molecular mechanisms in renal cell carcinoma (RCC) pathogenesis remain elusive.

**Methods:**

RT-PCR and Western blot were used to detect *PLCD1* expression in RCC cell lines and normal tissues. Bisulfite treatment, MSP and BGS were utilized to explore the CpG methylation status of *PLCD1* promoter. Online databases were analyzed for the association between *PLCD1* expression/methylation and patient survival. In vitro experiments including CCK8, colony formation, wound-healing, transwell migration and invasion, immunofluorescence and flow cytometry assays were performed to evaluate tumor cell behavior. Luciferase assay and Western blot were used to examine effect of *PLCD1* on WNT/β‐catenin and EGFR‐FAK-ERK signaling.

**Results:**

We found that *PLCD1* was widely expressed in multiple adult normal tissues including kidney, but frequently downregulated or silenced in RCC due to its promoter CpG methylation. Restoration of *PLCD1* expression inhibited the viability, migration and induced G2/M cell cycle arrest and apoptosis in RCC cells. *PLCD1* restoration led to the inhibition of signaling activation of WNT/β-catenin and EGFR-FAK-ERK pathways, and the EMT program of RCC cells.

**Conclusions:**

Our results demonstrate that *PLCD1* is a potent tumor suppressor frequently inactivated by promoter methylation in RCC and exerts its tumor suppressive functions via suppressing WNT/β‐catenin and EGFR‐FAK-ERK signaling. These findings establish *PLCD1* as a promising prognostic biomarker and treatment target for RCC.

**Supplementary Information:**

The online version contains supplementary material available at 10.1186/s13148-023-01448-2.

## Highlights

- *PLCD1* is methylated and downregulated in RCC and functions as a tumor suppressor gene
- Inactivation of *PLCD1* contributes to RCC tumorigenesis.
- *PLCD1* inhibits WNT/β-catenin and EGFR-FAK-ERK signaling in RCC.

**Supplementary Information:**

The online version contains supplementary material available at 10.1186/s13148-023-01448-2.

## Introduction

Kidney cancer, with renal cell carcinoma (RCC) accounting for 90% of the cases, is the most frequent malignancy of the urinary system [1]. RCC is a heterogeneous disease, comprising three main subtypes: kidney renal clear cell carcinoma (KIRC, or ccRCC), renal papillary cell carcinoma (KIRP) and kidney chromophobe carcinoma (KICH). Annually, approximately 430,000 new cases and 180,000 fatalities of RCC occur globally [2]. RCC is frequently discovered coincidentally during imaging for other health concerns, often at the late stage, with − 30% of patients already developed metastases at the time of diagnosis. RCC is primarily surgically treated and has a poor 5-year survival rate, with − 25% of patients reporting recurrence [3]. Thus, deeper understanding of the molecular pathogenesis of RCC is critical to develop efficient diagnostic markers and treatment strategy.

Epigenetic alterations, including promoter CpG methylation and histone modifications, are critically involved in multi carcinogenesis through silencing tumor suppressor genes (TSGs) and activating oncogenes [4, 5]. Due to the frequent loss of heterozygosity (LOH) at chromosome 3p21.3 in multiple cancers including esophageal squamous cell carcinoma (ESCC), lung cancer as well as nasopharyngeal carcinoma (NPC) [6], the 3p22-21.3 region is considered as a classic TSG locus. We and others previously discovered that several 3p22-21.3 genes including *RASSF1A, BLU, DLEC1, ZMYND10* and *PLCD1* were frequently methylated in various cancers [6–8].

Phospholipase C delta 1 (*PLCD1*), located at 3p22.2, is regarded as the basic isoform of PLC family members and belongs to the PLCδ subgroup [9]. *PLCD1* encodes an enzyme involved in calcium homeostasis, energy metabolism, intracellular transport and signal transduction. Silence of *PLCD1* by promoter CpG methylation has been described in a few epithelial and hematological malignancies, including gastric cancer (GsCa), ESCC, colorectal cancer (CRC), breast cancer (BrCa), pancreatic cancer (PAAD), chronic myeloid leukemia (CML) and non-small cell lung cancer (NSCLC) [10–14]. However, its relevance to RCC pathogenesis is unclear.

In this study, we examined the expression level and methylation status of *PLCD1* in RCC and further explored their relationship in patient tissues. Effects of ectopic *PLCD1* expression on RCC cell behavior and its underlying mechanisms were also evaluated. Our results demonstrate that *PLCD1* is a potent TSG disrupted by CpG methylation and closely involved in RCC pathogenesis and development.

## Methods

### Cell lines, normal tissue samples and RCC cell line samples

RCC cell lines including HH244, A498, Caki-2, RCC98, 786-O and HEK293T cells were obtained from American Type Culture Collection and maintained in RPMI-1640 or DMEM medium with 10% fetal bovine serum (FBS), 1% penicillin/streptomycin at 37 ℃ in a humidified atmosphere supplemented with 5% CO_2_. RNA samples of human normal adult tissues and RCC cell line panel were purchased from Stratagene (La Jolla, CA) or Millipore (Chemicon, Billerica, MA).

### Databases

*PLCD1* mRNA expression and protein expression of tumor and adjacent normal tissues were analyzed by GENT2 (GENT2 (appex.kr)), UALCAN (UALCAN (uab.edu)) and TIMER2.0 (TIMER2.0 (comp genomics.org))databases [15–17]. Human Protein Atlas version 20.1. (https://www.proteinatlas.org) was used to describe the *PLCD1* protein expression of various types of tissues. *PLCD1* genetic alteration rate in RCC patients was studied in cBioportal database (cBioPortal for Cancer Genomics), and mutation rate in TCGA samples was evaluated in TIMER2.0 database [17, 18]. *PLCD1* methylation status and its correlation with overall survival were analyzed on EWAS data hub (https://ngdc.cncb.ac.cn/ewas/datahub/index) [19, 20]. TIMER2.0 database was also employed to clarify the association between *PLCD1* expression and patients’ overall survival.

### Reverse transcription-polymerase chain reaction (RT-PCR) analysis

RT-PCR analysis for gene expression was done using GoTaq (Promega, Madison, WI). For *PLCD1* (F: 5’-TGTCGCTACTCAAGTGAGTC, R: 5’-AGTCCTCCTGCAACTTGTAG), the following program was conducted for 32 cycles: 95 °C for 30 s, 55 °C for 30 s and 72 °C for 30 s. *GAPDH* (*GAPDH*-F: 5’-GATGACCTTGCCCACAGCCT, *GAPDH*-R: 5’-ATCTCTGCCCCCTCTGCTGA) was used as a reference, and its reaction condition for 23 cycles was: 95 °C for 30 s, 55 °C for 30 s and 72 °C for 30 s.

### Bisulfite treatment and assessment of promoter methylation

DNA bisulfite modification and methylation-specific PCR (MSP), as well as bisulfite genomic sequencing (BGS), were used to detect promoter methylation. DNA extraction was achieved using DNA extraction kit (Qiagen, Hilden, Germany), and then, the extracted DNA was subjected to bisulfite treatment described previously [21]. After bisulfite treatment, unmethylated cytosines are converted into uracil, while methylated cytosine remains unchanged. We used allele-specific methylated (M) and unmethylated (U) primers for MSP amplification, and MSP products were analyzed by electrophoresis with 1.8% agarose gel. The amplified BGS products with BGS primers were purified and cloned into pCR4-TOPO cloning vector, with 6–8 colonies randomly selected for sequencing by Beijing Genomics institution (BGI). Primers used: MSP: m11: 5’-AATGATAGGGTTCGCGGTTC, m2: 5’-CCCGAACCAACGAACGCG, u1: 5’-GTAATGATAGGGTTTGTGGTTT, u2: 5’-CTAACCCAAACCAACAAACACA; BGS1: 5’-GTATTTTTGGGGTTAGAAATT, BGS4: 5’-AAAAACAAAACTAAAAACCC.

### 5-Aza-2′-deoxycytidine (5-Aza) and trichostatin A (TSA) treatment

Cell lines (A498, Caki-2, RCC98, HH244, and 786-O) were freshly seeded at a density of 1 × 10^5^ cells/ml and allowed to grow in T175 flasks overnight. Fresh medium with 10 µM 5-Aza (Sigma-Aldrich, St Louis, MO) was changed every 24 h for 3 days and then harvested; or further treated with 100 nM trichostatin A (TSA) for additional 24 h. Cells were harvested for RNA and DNA extraction. The dosages of 5-Aza and TSA were decided based on its dose–response effect on cell viability and demethylation effect previously assessed.

### Colony formation assay

For colony formation analysis, A498 and HH244 cells in 6-well plates (2 × 10^5^ cells/well) were transfected with pcDNA3.1( +)-*PLCD1*-V5/His plasmid or the control vector (2.5 μg/well), using Lipofectamine™ 3000 Reagent (Invitrogen). 24 h post-transfection, cells were harvested by trypsinization and centrifugation and plated on 6-well plates at a suitable density. The cells were maintained with G418 (0.4 mg/mL) and stably transfected cells selected for 10–12 days with selection media changed every 2 days. Surviving colonies (> 50 cells per colony) were stained using Gentian Violet (ICM Pharma) and counted. Assays were performed for three times, in triplicate wells.

### Cell counting kit-8 (CCK-8) assay

Cell proliferation was evaluated by CCK-8 assay. A498 and HH244 cells in 6-well plates (2 × 10^5^/well) were transfected with pcDNA3.1( +)-*PLCD1*-V5/His plasmid or control vector (2.5 μg each well), using Lipofectamine™ 3000 Reagent (Invitrogen). After 24 h, cells were obtained and plated into 96-well plates at a density of 2000 cells/well. Cell proliferation rates were evaluated by using CCK-8 assay (MCE, HY-K0301). Absorbance at 450 nm was measured at designated time point by spectrophotometer. All assays were conducted for three times in triplicates.

### Transwell migration and invasion analyses

Cell migration and invasion were examined using transwell migration and Matrigel invasion assays. Migration assay was done on 24-well transwell inserts (8-um-pore filters, BD Biosciences). Matrigel invasion assays were done on 24-well plates containing Matrigel (8.0 Micron, Corning). After 24 h of transfection, RCC cells were harvested, resuspended in RPMI-1640 medium (serum-free), with 2.5 × 10^4^ cells seeded into the upper chamber. Then, 10% FBS-supplemented complete medium was added into the lower chambers. After 24 h of incubation, the inserts were fixed in methanol for 25 mins, stained using crystal violet, and non-migratory and non-invasive cells were eliminated with cotton-tipped swabs. Cell numbers were counted for different fields using a microscope, with the mean number of cells per field determined. Analyses were conducted independently for 3 times.

### Wound-healing assay

Briefly, transfected cells (2.5–3.5 × 10^5^) were seeded in 6-well plates. Careful scratching of the monolayer was done using sterile yellow tips followed by washing using phosphate-buffered saline (PBS). A light microscope was used to observe and measure the scratch width. All assays were independently repeated 3 times.

### Cell cycle and apoptosis analyses

Cell cycle and apoptosis were evaluated by flow cytometry. RCC cells (A498 and HH244) were transfected with pcDNA3.1( +)-*PLCD1*-V5/His plasmid or control vector, and washed with PBS before fixed with 70% ice-cold ethanol. For apoptosis, cells were stained with propidium iodide (PI) and Annexin V-FITC (BD Biosciences, Bedford, MA). Cells were stained with PI (Sigma) for cell cycle assessment. Both were sorted by BD Accuri C6 (BD Biosciences, Bedford, MA) as instructed by the manufacturer. Cell cycle data were analyzed using Modfit 3.0, while apoptosis assay finding was evaluated using BD Accuri C6 Software. Assays were done in triplicates.

### Dual-luciferase reporter assay

This assay was conducted to evaluate transcriptional activity. To identify *PLCD1*-modulated signaling pathways, several luciferase reporters for signaling pathways were assessed in HEK293T, A498 and HH244 cells, following exogenous expression of *PLCD1*. CCND1p, p53-bs, MMP7p, C-mycp, PAI-1p, NF-κB-bs, AP1-bs, GRR5, SRE (Stratagene), as well as TOPflash plasmids (gifts from Prof. Christof Niehrs, DKFZ, Heidelberg), were individually co-transfected into cells along with Renilla and pcDNA3.1( +)-*PLCD1*-V5/His or pcDNA3.1( +). Cells were harvested after 48 h, and luciferase assay was performed using the Dual-Luciferase® reporter assay system (Promega). At least 3 independent experiments with triplicate wells were done.

### Western blot assay

Cell lysis was done in ice-cold RIPA lysis buffer supplemented with protease and phosphatase inhibitors. Then, proteins were resolved by SDS-PAGE and electroblotted onto Hybond-P membranes (Amersham). Membranes were blocked using Blotting-Grade Blocker (Bio-Rad, cat# 1,706,404) followed by incubation with primary antibody overnight (4℃), and then secondary antibody for 1 h at room temperature. Signal was then developed by ECL™ Detection Reagents (Amersham Biosciences) and quantitated with ImageJ. Antibodies used are: anti-mouse IgG-HRP (DAKO, P0161), anti-rabbit IgG-HRP (DAKO, P0448), GAPDH (Millipore, MAB374), Anti-V5-Tag (Invitrogen, R96025), E-cadherin (CST, 4065), vimentin (Sigma: V6630), Twist (Santa Cruz, sc-15393), MMP7 (Thermo Fisher, MS-813-P0), Total EGFR (CST: 54,359), p-EGFR (CST: 3777), Total FAK (CST: 71,433), p-FAK (Tyr397) (CST: 8556), Total SRC (CST: 2191), p-SRC (CST: 59,548), Total β-catenin (CST: 59,548), active β-catenin (Millipore, 05,665), p-β-catenin (Ser552) (CST: 5651), c-Myc (CST: 18,583), cyclinD1 (CST: 55,506), p-ERK1/2 (CST: 9101), Total ERK1/2 (CST: 4695), Cleaved-Caspase3 (CST: 9661), Cleaved-PARP (CST: 9541), Caspase 3 (CST:9504), PARP (CST: 9532), Bax (CST:2772), Bcl-2 (CST: 2872).

### Immunofluorescence

Cells were plated on cover slips in 6-well plates (80,000 cells/well). After transfection for 48 h, cells were fixed in paraformaldehyde (PFA) (4%), permeabilized using Triton X-100 (0.1%), followed by blocking with normal goat serum (DAKO, X0907). Cells on cover slips were incubated with primary and secondary antibodies (Anti-rabbit IgG-Alexa Fluor 488-F(ab')2 antibody: Thermo Fisher, A-11070) successively, and nucleus was counterstained with 4′,6-diamidino-2-phenylindole (DAPI). For phalloidin staining, cells were incubated with rhodamine phalloidin for 1 h at room temperature after permeabilization. Cover slips were mounted with DABCO and then observed by immunofluorescence microscopy.

### Statistical analysis

Data analyses were performed using SPSS version 22.0. Fisher exact test, two-tailed t test or chi-square test were used to evaluate *p* values. *p* < 0.05 denoted significance.

## Results

### *PLCD1* is silenced by promoter CpG methylation in RCC cell lines

**Fig. 1:**
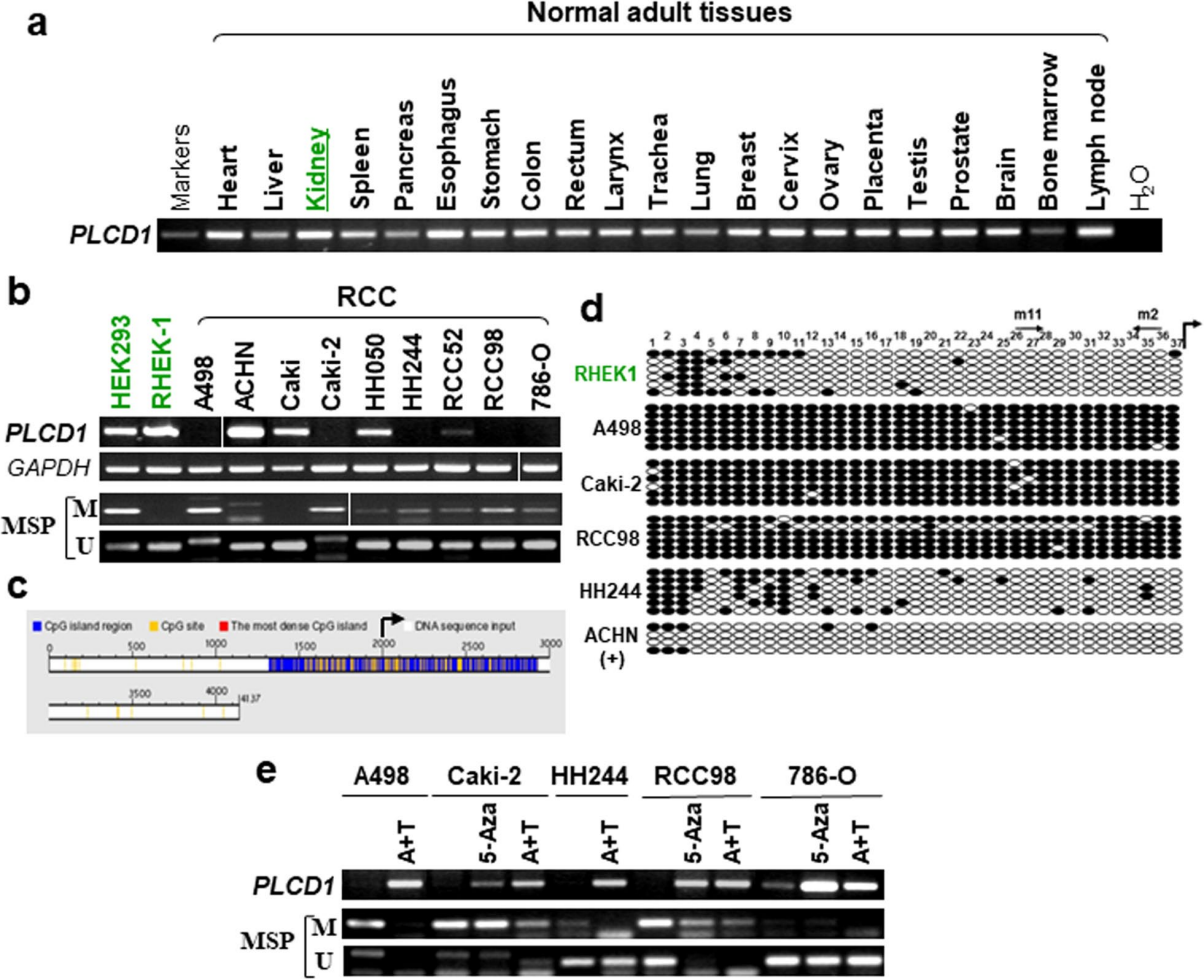
*PLCD1* expression and promoter CpG methylation analysis. **a** *PLCD1* expression profile across human adult normal tissues examined by RT-PCR assay. **b** Expression of *PLCD1* in RCC cell lines and human normal embryonic kidney cells, *GAPDH* was used as a reference gene. MSP was used to assess *PLCD1* promoter methylation in RCC cell lines and immortalized epithelial cells. M: methylated, U: unmethylated. **c** CpG island at *PLCD1* promoter identified by Database of CpG islands and Analytical Tool (DBCAT). Bent-tailed arrow denotes transcriptional start site. **d** BGS result confirmed *PLCD1* promoter methylation in RHEK-1, A498, Caki-2, RCC98, ACHN and HH244 cells. Full circle represents methylated site, and open circle is unmethylated site. **e** *PLCD1* restoration by demethylation treatment using 5-Aza alone or 5-Aza combined with TSA (A + T) in tumor cell lines, accompanied by promoter demethylation. *RCC* Renal cell carcinoma

We first evaluated the expression profile of *PLCD1* using RT-PCR and bioinformatics analysis. RT-PCR analysis conducted in a panel of normal adult tissues showed that *PLCD1* was widely expressed in multiple normal adult tissues (Fig. 1a). Likewise, Protein Atlas analysis showed that *PLCD1* protein was expressed among an array of human normal tissues, including kidney (Additional file 1: Fig. S1a) [22]. We then detected *PLCD1* mRNA expression in RCC cell lines and found that *PLCD1* was robustly expressed in HEK293 and RHEK-1, which were immortalized normal kidney cell lines, but silenced or downregulated in 6/9 RCC cell lines (Fig. 1b). According to the criteria in the Database of CpG islands and Analytical Tool (DBCAT): minimal island size > 200 bp, CG content > 50% and Obs/Exp > 0.6, a CpG island (CGI) was identified at the *PLCD1* promoter (Fig. 1c). Thus, the role of promoter CpG methylation in *PLCD1* silencing raised our interest. We performed MSP with methylation-specific and unmethylation-specific primers and found that *PLCD1* was methylated in 7/9 RCC cell lines, correlated with its mRNA suppression (Fig. 1b). To verify the MSP findings, we carried out BGS to evaluate the methylation status of 37 CpG sites within the *PLCD1* promoter. There were densely methylated alleles in *PLCD1*-silenced cells, such as A498 (99% CpG sites methylated), Caki-2 (99% CpG sites methylated) and RCC98 (90% CpG sites methylated). Consistently, more unmethylated alleles were detected in RHEK-1 and ACHN cells (Fig. 1d). RCC cell lines with *PLCD1* methylation/downregulation were further treated with DNA methyltransferase inhibitor, 5-Aza, either alone or in combination with a histone deacetylase (HDAC) inhibitor, trichostatin A. Cell viability of 80–90% was observed after exposure to this concentration (10 µM) of 5-Aza for 3 days. Results showed that *PLCD1* mRNA level could be restored by demethylation treatment, accompanied by promoter allele demethylation (Fig. 1e).

### CpG methylation predominantly accounts for *PLCD1* downregulation, and correlated with survival in patients

**Fig. 2:**
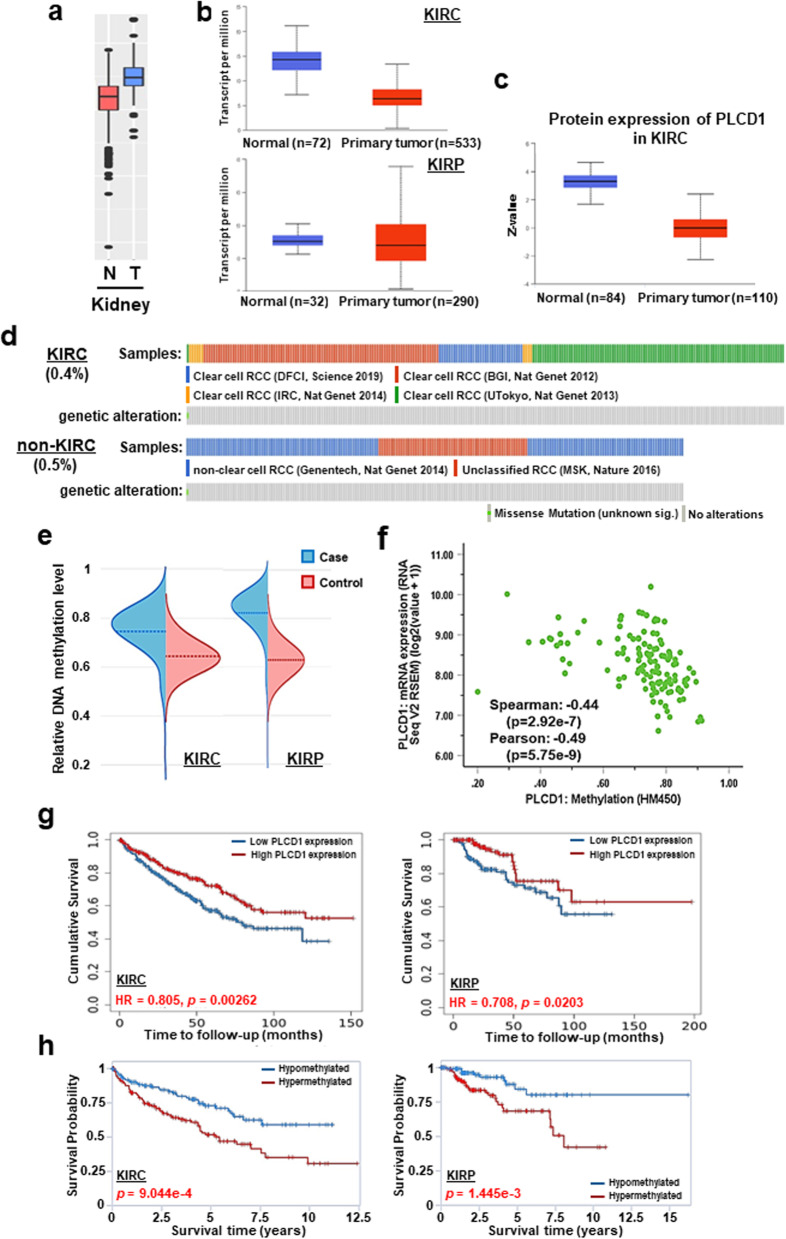
Bioinformatics analysis of *PLCD1* gene in tissues. **a** Comparison of *PLCD1* expression level between normal and tumor renal samples through GENT2 database. N: normal, T: tumor. **b** mRNA expression of *PLCD1* based on sample types in KIRC (upper lane) and KIRP (bottom lane) patients. **c** *PLCD1* proteomic expression profile in normal and primary tumor samples from KIRC patients. The data of **b** and **c** were retrieved from UALCAN database. **d** Oncoprint plots showing genetic alteration rates of *PLCD1* in KIRC and renal non-clear cell carcinoma patients from cBioportal database. **e** DNA methylation-level comparison between case and control samples from KIRC (left one) and KIRP (right one) patients through EWAS Data Hub. **f** Scatter diagram depicting the negative correlation between *PLCD1* methylation and mRNA expression. The raw data was extracted from EWAS Data Hub. **g** Kaplan–Meier curves illustrating the association of *PLCD1* expression and methylation **h** with the overall survival of KIRC (left lane) and KIRP (right lane) patients, respectively. The plots of **g** were captured from TIMER 2.0 database and **h** from EWAS Data Hub. *KIRC* Kidney renal clear cell carcinoma; *KIRP* kidney renal papillary cell carcinoma

Next, we investigated the relationship between expression and methylation of *PLCD1* in patient tissues. By analyzing theTIMER2.0 database, we found differential expression of *PLCD1* between tumor and adjacent tissues across TCGA tumors. Relative to adjacent normal tissues, *PLCD1* exhibited significant lower expression in many types of cancers, including RCC (Fig. 2a, Additional file 1: S1b). This distinctive expression model could be observed in different subtypes of RCC, both KIRC and KIRP (Fig. 2b). Additionally, compared with adjacent normal tissues, *PLCD1* displayed a reduced protein expression in primary KIRC analyzed by UALCAN database (Fig. 2c). With the consistent decrease of *PLCD1* expression in RCC, we proceeded to examine the contribution of its genetic alteration. Data from TIMER2.0 database showed that *PLCD1* manifested rare mutations across a spectrum of TCGA cancers, with only 0.54% (2/370) in KIRC patients (Additional file 1: Fig. S1c). Apart from TCGA patients, we analyzed *PLCD1* alteration rate in patients involved in other reported studies in the cBioportal database. Similar low mutation rates of *PLCD1* was found, with 0.4% (1/249) in KIRC and 0.5% (1/207) in non-KIRC renal carcinoma (Fig. 2d). Therefore, methylation appears to be the main driver of *PLCD1* disruption in cancer patients. This hypothesis is also supported by information from EWAS Data Hub, which revealed that *PLCD1* methylation level in tumors was significantly higher than that in adjacent normal tissues (Fig. 2e). By further analyzing 3611 patient samples in 17 kidney related studies from the cBioportal database, we found an inverse correlation between *PLCD1* methylation and its mRNA expression in 124 cases (Fig. 2f). Moreover, *PLCD1* downregulation is of clinical significance, substantially associated with tumor stage and distant metastasis of KIRC and KIRP patients in TCGA database (Tables 1, 2). Moreover, we found that RCC patients with higher *PLCD1* expression, including KIRC and KIRP, had a better prognosis in the TIMER2.0 database (Fig. 2g). Furthermore, *PLCD1* methylation was linked with worse overall survival of RCC patients (Fig. 2h). Taken together, our findings suggest that *PLCD1* methylation was the main cause of its silencing and could be a prognostic biomarker.

**Table 1:** Association between PLCD1 expression and clinicopathological parameters of KIRC patients in TCGA

Characteristic	Low expression of PLCD1	High expression of PLCD1	*p* value
*n*	269	270	
T stage, *n* (%)			< 0.001
T1	118 (21.9%)	160 (29.7%)	
T2	33 (6.1%)	38 (7.1%)	
T3	111 (20.6%)	68 (12.6%)	
T4	7 (1.3%)	4 (0.7%)	
N stage, *n* (%)			0.593
N0	126 (49%)	115 (44.7%)	
N1	10 (3.9%)	6 (2.3%)	
M stage, *n* (%)			0.001
M0	209 (41.3%)	219 (43.3%)	
M1	54 (10.7%)	24 (4.7%)	
Age, median (IQR)	61 (53, 71)	60 (51, 68.75)	0.155

**Table 2:** Association between PLCD1 expression and clinicopathological parameters of KIRP patients in TCGA

Characteristic	Low expression of PLCD1	High expression of PLCD1	*p* value
*n*	144	145	
Pathologic T stage, *n* (%)			< 0.001
T1	79 (27.5%)	114 (39.7%)	
T2	18 (6.3%)	15 (5.2%)	
T3	45 (15.7%)	14 (4.9%)	
T4	1 (0.3%)	1 (0.3%)	
Pathologic *N* stage, *n* (%)			0.041
N0	32 (41.6%)	17 (22.1%)	
N1	22 (28.6%)	2 (2.6%)	
N2	3 (3.9%)	1 (1.3%)	
Pathologic M stage, *n* (%)			0.004
M0	49 (47.1%)	46 (44.2%)	
M1	9 (8.7%)	0 (0%)	
Age, median (IQR)	61 (54, 69.25)	62 (53, 73)	0.268

### *PLCD1* restoration inhibits RCC cell proliferation, migration and invasion

**Fig. 3:**
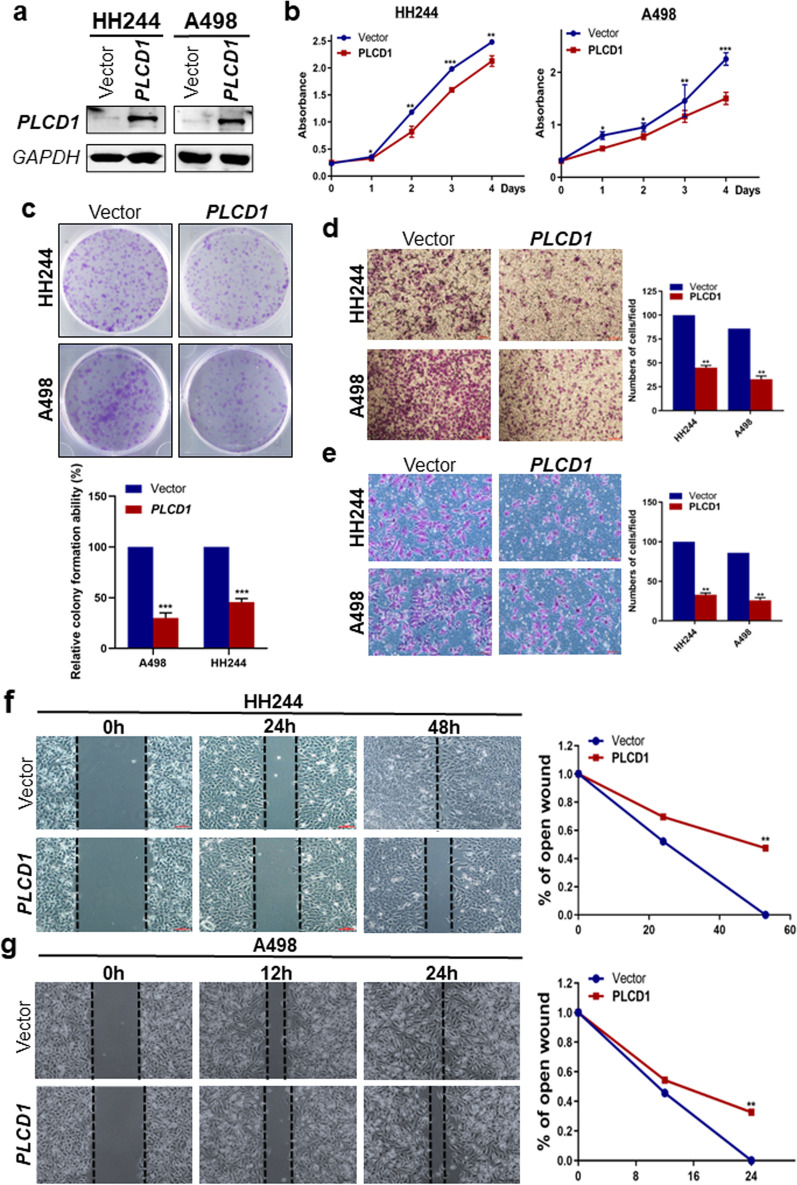
Effect of *PLCD1* expression on cell viability, migration and invasion in A498 and HH244 cells. **a** Western blot analysis confirming the ectopic *PLCD1* expression in A498 and HH244 cells by transfecting with the pcDNA3.1-*PLCD1* plasmid. **b** Cell viability was evaluated using Cell Counting Kit-8 (CCK-8) assay, and absorbance at 450 nm was measured to reveal the proliferation rates at each time point. **c** Representative colony formation assays performing in vector- or *PLCD1*-expressing HH244 and A498 cells. The histogram was quantitative analysis of colony-formed numbers in each group. **d** Representative results of transwell migration and invasion **e** assays in HH244 and A498 cells. Migratory or invasive cells on the underside membrane of the chambers were fixed and stained with crystal violet and then counted under a light microscope. **f**, **g** Wound-healing assay in RCC cell lines, HH244 **f** and A498 **g** cells, transfected with vector alone or with pcDNA3.1-*PLCD1* plasmid. Images were taken at 0, 12, 24 and 48 h by microscopy. The percentage of open wound over time was analyzed with line charts. The symbols *, ** and *** in these figures indicated *p* < 0.05, *p* < 0.01 and *p* < 0.001, respectively

We moved on to examine the biological roles of *PLCD1* in RCC cells. Ectopic *PLCD1* expression was confirmed by Western Blot (Fig. 3a). Analysis of RCC cells viability using CCK-8 and colony formation assays revealed that ectopic *PLCD1* expression dramatically reduced the proliferation rate of RCC cells. The numbers and size of colonies formed in *PLCD1*-expressing cells were reduced, relative to the control group (Fig. 3b–c).

To further assess the functions of *PLCD1*, we performed wound-healing, transwell migration and invasion assays. *PLCD1* was found to have an inhibitory effect on both migration and invasion ability of RCC cells. RCC cells transfected with *PLCD1* migrated and invaded at a slower rate than those transfected with empty vector (Fig. 3d–e). The efficiency of closing the open wound was greatly suppressed in *PLCD1*-expressing cells, compared to vector group (Fig. 3f, g).

### *PLCD1* re-expression attenuates EMT in A498 and HH244 cells

**Fig. 4:**
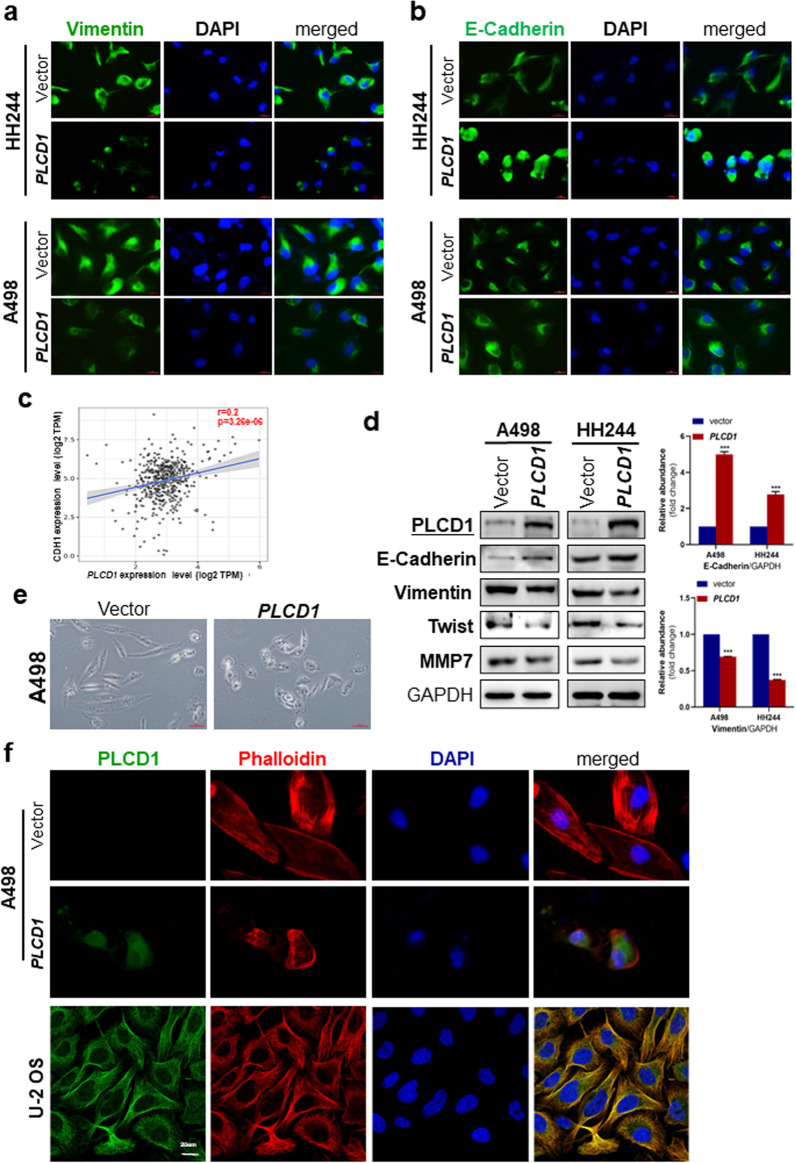
*PLCD1* overexpression attenuated EMT in A498 and HH244 cells. Immunofluorescence analysis of vimentin **a** and E-cadherin **b** in RCC cells transfected with *PLCD1* plasmid or vector only. Cell nucleus were visualized by DAPI counterstaining. **c** Positive association between *PLCD1* and *CDH1* expression level analyzed by TIMER2.0 website. **d** Western blot analysis of E-cadherin, vimentin, Twist, MMP7 and *PLCD1* with GAPDH as loading control. Graphs represent quantification of bands intensity with fold change compared with controls. **e** Morphological changes of A498 cells overexpressing *PLCD1* relative to cells transfected with vector only. Scale bar: 45um. **f** Subcellular localization of *PLCD1* and phalloidin staining. U-2 OS is a human osteosarcoma cell. The images were picked from Human Protein Atlas. Scale bar: 20um. CDH1: Cadherin1

Epithelial–mesenchymal transition (EMT) is critical in cancer metastasis, a process in which epithelial cells obtain mesenchymal characteristics like cell motility and migration [23]. Based on the influence of *PLCD1* expression on distant metastasis of RCC patients, we examined the effect of *PLCD1* expression on EMT. We conducted immunofluorescence assay to check the cellular location and expression of E-cadherin and vimentin. Results showed that compared to control group, *PLCD1*-transfected cells had higher E-cadherin and lower vimentin fluorescence (Fig. 4a–b). Consistent with this, we discovered that *PLCD1* expression was positively associated with *CDH1* expression in KIRC patients in TCGA database (Fig. 4c). Western blot assays showed that *PLCD1* ectopic expression upregulated the epithelial marker E-cadherin and suppressed the expression of mesenchymal markers (vimentin, Twist, MMP7) in A498 and HH244 cells, indicating that *PLCD1* inhibited EMT (Fig. 4d). In accordance with this, after transfection with *PLCD1*-expressing plasmid, A498 cell morphology changed from a long and spindly to a fried-egg morphology (Fig. 4e). Confocal microscopy showed that *PLCD1* is mainly localized in microtubules in U-2 OS cells, which is an important part of cytoskeleton and provides shape maintenance (Fig. 4f). The subcellular localization of *PLCD1* could partly explain its functions in cytoskeleton remodeling and epithelial morphology regulation. This was further supported by fluorescence staining with rhodamine phalloidin in A498 cells, in which actin filaments distributed along the elongated and well-spread cells in vector group, while clustered in *PLCD1*-expressing cells. These results demonstrate that *PLCD1* suppresses the EMT program and further inhibits the migration and invasion ability of RCC cells.

### Ectopic *PLCD1* induces G2/M cell cycle arrest and RCC cell apoptosis

**Fig. 5:**
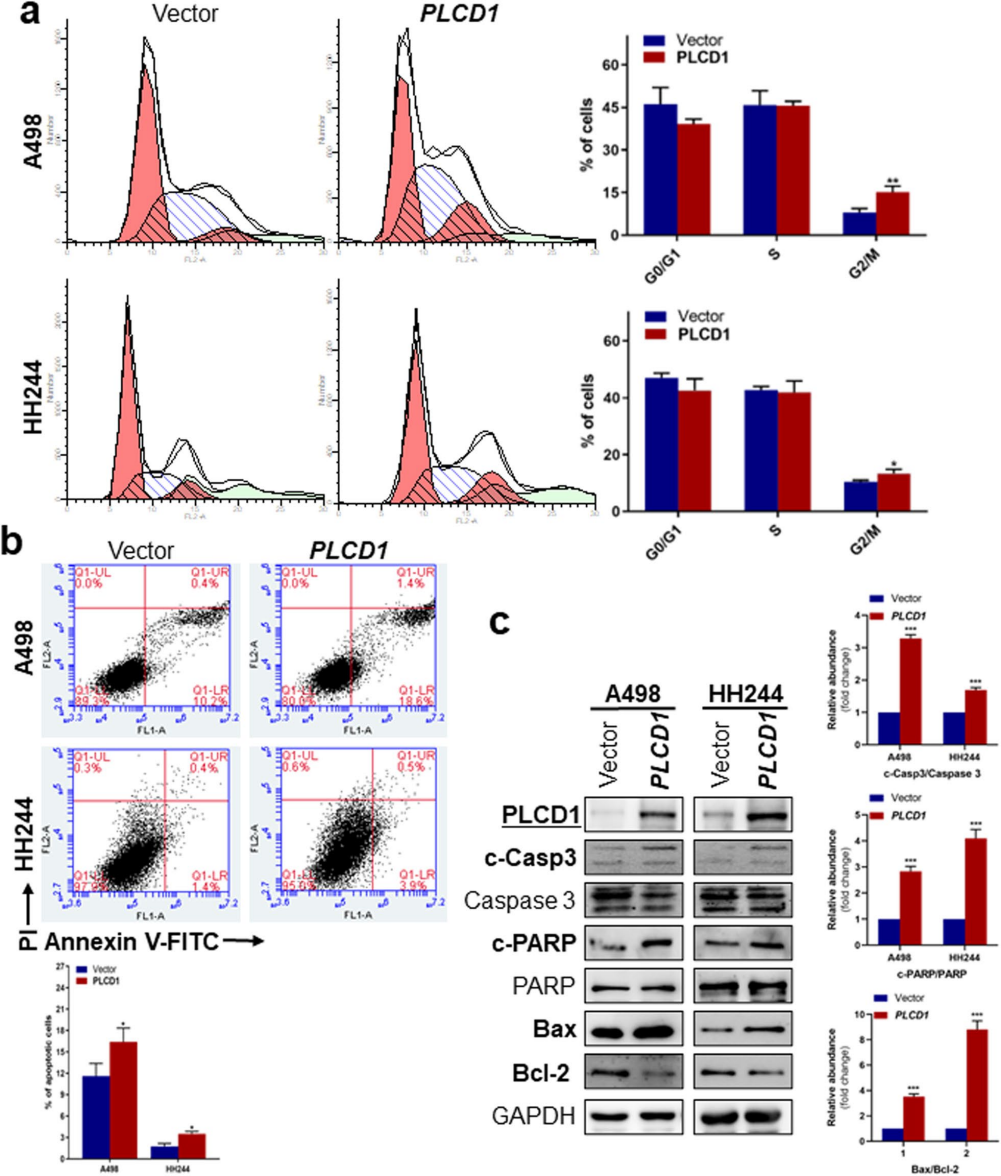
Cell cycle and apoptosis analyses of *PLCD1*. **a** Effects of *PLCD1* on the cell cycle distribution. The bar graphs described the percentage of cells in G0/G1, S and G2/M phases. **b** Cell apoptosis was analyzed by propidium iodide (PI) and Annexin V-FITC staining and assessed using flow cytometry. The percentage of early apoptotic cells in each group was shown in the quantification histogram. **c** Immunoblotting analysis of apoptosis-related markers, cleaved-caspase 3 (cleaved-Casp3), caspase 3, cleaved-PARP, PARP, Bax and Bcl-2. Relative protein-level ratio quantified by ImageJ was shown as histogram

Next, we used flow cytometry to analyze cell cycle and apoptosis status of RCC cells. We found that *PLCD1*-expressing cells exhibited significant increase in cells at G2/M phases post-transfection (A498: control: 8.01%, *PLCD1*: 15.20%, *p* = 0.0072; HH244: control: 10.34%; *PLCD1*: 15.69%, *p* = 0.0030), relative to control cells, but with no statistic difference in S phase cells (A498: control: 42.07%*, PLCD1*: 45.66%, *p* = 0.9316; HH244: control: 42.75%; *PLCD1*: 29.44%, *p* = 0.0605) and G0/G1 phase (A498: control: 49.64%, *PLCD1*: 41.08%, *p* = 0.1211; HH244: control: 46.91%; *PLCD1*: 54.86%, *p* = 0.1943) (Fig. 5a). Apoptosis analysis revealed that in contrast to vector control, *PLCD1*-transfected cells had a higher proportion of early apoptotic cells (A498: 11.6 ± 1.78% vs 16.4 ± 1.93%, *p* < 0.05, HH244: 1.73 ± 0.42% vs 3.5 ± 0.36%,* p* < 0.05) (Fig. 5b). Western blot of apoptosis biomarkers revealed that relative to controls, A498 and HH244 cells transfected with *PLCD1* plasmids had significantly higher ratio of cleaved-Caspase 3/caspase 3 and cleaved-PARP/PARP levels (Fig. 5c). Consistently, pro-apoptotic Bax level was elevated and anti-apoptotic protein Bcl-2 was decreased, accompanied by a significantly upregulated Bax/Bcl-2 ratio in *PLCD1*-expressed cells (Fig. 5c). These findings showed that *PLCD1* triggered RCC cell cycle arrest in G2/M phase and induced apoptosis.

### *PLCD1* antagonizes WNT/β-catenin signaling in RCC cells

**Fig. 6:**
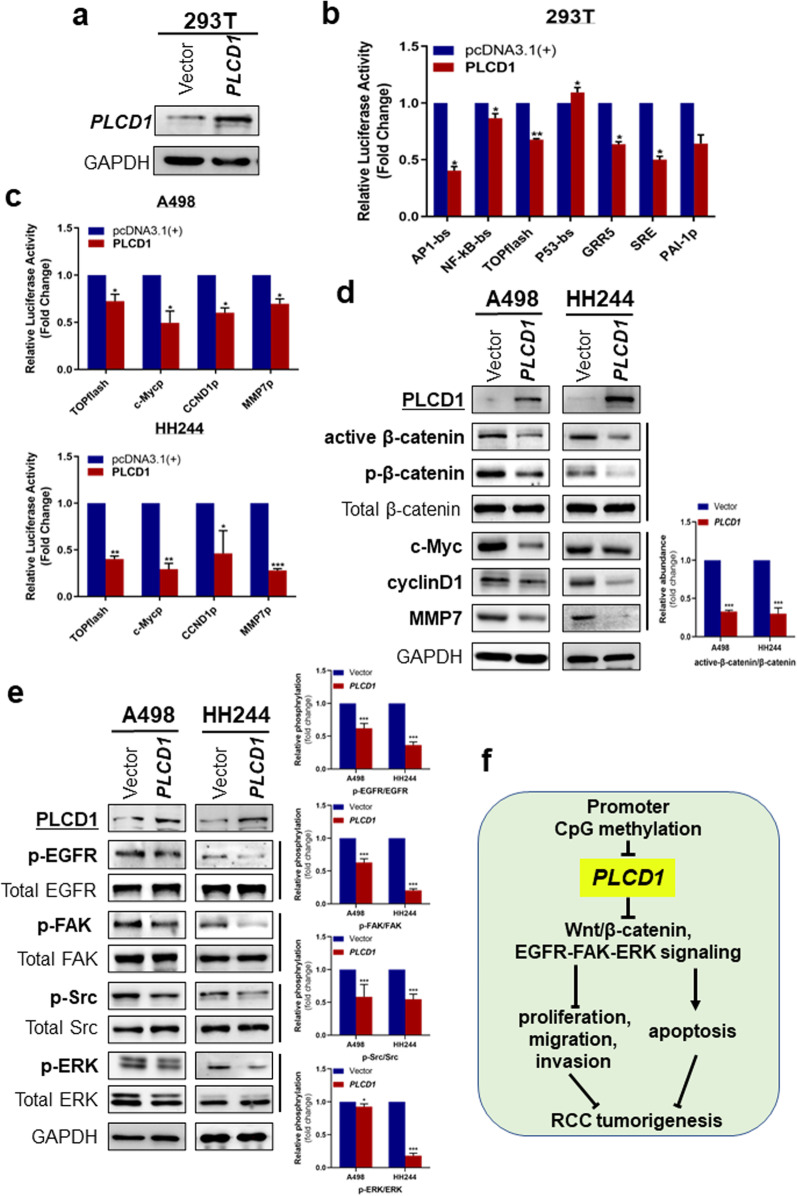
*PLCD1* ectopic expression inhibited oncogenic signaling. **a** Experimentally expressed *PLCD1* in 293 T cells was confirmed by Western blot. **b** Activity of several oncogenic signaling reporters were evaluated using dual-luciferase reporters assay in 293 T cells. **b** TCF activity and *CCND1*, *c-Myc* and *MMP7* promoter reporter activities in *PLCD1*-overexpressing A498 (upper) and HH244 (bottom) cells. **d** Western blot analyses of total β-catenin, p-β-catenin (Ser552), c-Myc, active β-catenin, cyclinD1, MMP7, *PLCD1* and GAPDH. **e** Western blot analyses of EGFR, ERK1/2, Src, FAK and *PLCD1*, with GAPDH as internal control. Histograms indicated quantification of the phosphorylated protein normalized to corresponding total protein level. **f** Schematic diagram of roles and mechanisms of *PLCD1* in RCC tumorigenesis

To investigate the molecular mechanisms underlying the role of *PLCD1* in RCC development, we employed dual-luciferase reporter assays to determine its influence on various oncogenic pathways. Western blot was used to evaluate *PLCD1* expression in HEK239T cells (Fig. 6a). Ectopic *PLCD1* expression significantly suppressed transcriptional activities for AP1-bs, NF-κB-bs, TOPflash, GRR5 and SRE elements in 293 T cells (Fig. 6b). TOPflash (TCF reporter plasmid), a reporter containing TCF binding sites, was used to detect canonical Wnt/β-catenin signaling [24]. Inspired by decreased TOPflash activity, we then mainly focused on Wnt/β-catenin signaling. *MMP7*, *c-Myc* as well as *CCND1* are the downstream target genes of Wnt/β-catenin signaling. Next, we tested the reporter activities of MMP7p, c-Mycp and CCND1p after transfection with *PLCD1*-plasmid. Data showed that *PLCD1* markedly suppressed the reporter activities as well as expressions of c-Myc, Cyclin D1 and MMP7 in RCC cells (Fig. 6c–d). Furthermore, active β-catenin and phospho-β-catenin (ser552) levels were decreased (Fig. 6d). Together, these findings suggest that *PLCD1* inhibited the activation of β-catenin and antagonized WNT/β-catenin signaling.

### *PLCD1* suppresses EGFR-FAK-ERK signaling in RCC cells

Epidermal growth factor receptor (EGFR), a member of ErbB family of receptor tyrosine kinase (RTK), is closely involved in human carcinogenesis [22]. Additionally, focal adhesion kinase (FAK) and steroid receptor coactivator (Src) often function as complex to mediate signaling required for tumor initiation and progression [25, 26]. Extracellular signal regulated kinase (ERK) cascade is a well-known downstream of FAK/Src-activated phosphorylation events, following the stimulation of vascular endothelial growth factor (VEGF), which leads to tumor angiogenesis [27]. In dual-luciferase reporter assays, we noted that the activity of SRE reporter was blocked after *PLCD1* restoration, suggesting that PLCD1 inhibits ERK signaling (Fig. 6b). By Western blot analysis, we found that relative to vector control, transfection with *PLCD1* plasmid reduced the levels of p-EGFR, p-Src, p-FAK (Tyr397) and p-ERK, but did not significantly change the level of their total proteins (Fig. 6e), demonstrating that *PLCD1* negatively regulates EGFR-FAK-ERK signaling in RCC cells.

## Discussion

RCC carcinogenesis is a complex and multi-step process, involving aberrant alterations of multiple cancer genes and cell signaling pathways which confers a serials of cell physiologic responses with regard to proliferation, migration, invasion and apoptosis. In the present study, we demonstrate that *PLCD1* exhibited low protein and mRNA levels in RCC. *PLCD1* downregulation was validated in RCC cell lines and tumors, correlated with promoter CpG methylation. We also validated the tumor suppressive roles of *PLCD1* with multiple biological assays, which is achieved by suppressing Wnt/β-catenin and EGFR-FAK-ERK signaling in RCC cells.

We detected *PLCD1* expression profile and found that it was downregulated or silenced in 6/9 of RCC cell lines. After demethylation treatment, *PLCD1* downregulated cell lines demonstrated considerable increase in *PLCD1* transcription, which confirms dominant role of promoter CpG methylation in *PLCD1* silencing. On the other hand, only rare mutations of *PLCD1* were observed in RCC tumor tissues, indicating that *PLCD1* silencing is the predominant type of *PLCD1* inactivation in RCC.

Our results demonstrate that *PLCD1* functions as a tumor suppressor in RCC cells, consistent with previous reports in other types of carcinomas. Previously, our laboratory identified the tumor inhibitory function of *PLCD1* in breast cancer by inducing cell cycle G2/M arrest [13]. In addition, *PLCD1* restoration was found to impede the proliferation, migration and invasion abilities of ESCC cells through modulating the Wnt/β-catenin pathway [14]. WNT/β-catenin signaling regulates the expression of numerous target genes, including *Axin-2*, *c-Myc*, *TCF-1*, *CCND1*, *MMP7* and *CD44* [28], thereby regulating stem cells pluripotency and controlling cell destiny during development and diseases. During tumorigenesis, WNT signaling is frequently inappropriately activated to facilitate malignant behavior, such as proliferation, migration and invasion [27]. Approximately half of human malignancies have been linked to aberrant activation of WNT/β-catenin signaling [29]. *PLCD1* has been shown to function as a TSG by inhibiting WNT/β-catenin in ESCC, BrCa and CRC [14, 30, 31], consistent with our findings that *PLCD1* inhibits active-β-catenin and downstream target gene transcription, including *MMP7*, *c-Myc* and *CCND1*, hence suppressing proliferation, migration and invasion of RCC cells.

EGFR is a critical regulator of a variety of cell biological processes, such as proliferation, division, differentiation and survival [32]. EGFR signaling is also explored for drug development and clinical management [33]. Erlotinib, an EGFR inhibitor, exhibits promising effect in advance RCC patients in undergoing clinical trials [34]. PLC family has been reported to display a negative correlation with EGFR expression in breast, pancreatic and ovarian cancers [35–38]. Herein, we found that ectopic *PLCD1* expression negatively regulated EGFR activation in RCC cells. Thus, we speculated the combination of epigenetic inhibitors and EGFR antagonists might achieve better therapeutic effects in RCC treatment. More clinical data should be collected to validate this hypothesis.

FAK/Src complex acts as intracellular nexus, transducing signals from integrin or tyrosine kinase receptors (TKRs), involving in different steps of neoplastic initiation and development [23, 38]. When activated by upstream stimulation, phosphorylation occurs at the tyrosine residue 397 (Y397) of FAK [39,40]. Then, Src is recruited to phospho-FAK and get phosphorylated and activate each other exponentially. ERK is one of the downstream effectors of FAK/Src signaling, which modulates EMT-related metastasis and VEGF-associated angiogenesis [41,42]. Currently, VEGFR inhibitors, such as bevacizumab, have become an important targeted therapy for malignant diseases [43]. In general, our results demonstrate that *PLCD1* expression inhibited EGFR phosphorylation and both activated form of FAK/Src and ERK. The intervention of *PLCD1* in EGFR-FAK-ERK signaling indicates its potential value as a treatment target for RCC patients in clinical practice.

## Conclusions

In this study, we identified *PLCD1* as a functional TSG downregulated by promoter CpG methylation in RCC, and it exerts tumor suppressor effects by blocking WNT/β-catenin and EGFR-FAK-ERK signaling. These findings establish *PLCD1* as a prognostic biomarker and treatment target for RCC.

## Supplementary Information

**Additional file 1**: Overview of *PLCD1* bioinformatics analysis. **a** Protein expression of *PLCD1* in 45 kinds of tissues through Human Protein Atlas. **b** Differential expression of *PLCD1* between tumor and adjacent tissues across TCGA tumors in TIMER2.0 database. **c** *PLCD1* mutation rates across TCGA tumors analyzed by TIMER 2.0 database.

## Data Availability

All datasets generated for this study are included in the article and supplementary materials.
