# Supplementary figures and images for: Phospholipase C delta 1 inhibits WNT/β‐catenin and EGFR-FAK-ERK signaling and is disrupted by promoter CpG methylation in renal cell carcinoma
Source: Clin Epigenetics. 2023 Feb 27;15:30. doi: 10.1186/s13148-023-01448-2 (PMC9972803; doi:10.1186/s13148-023-01448-2)

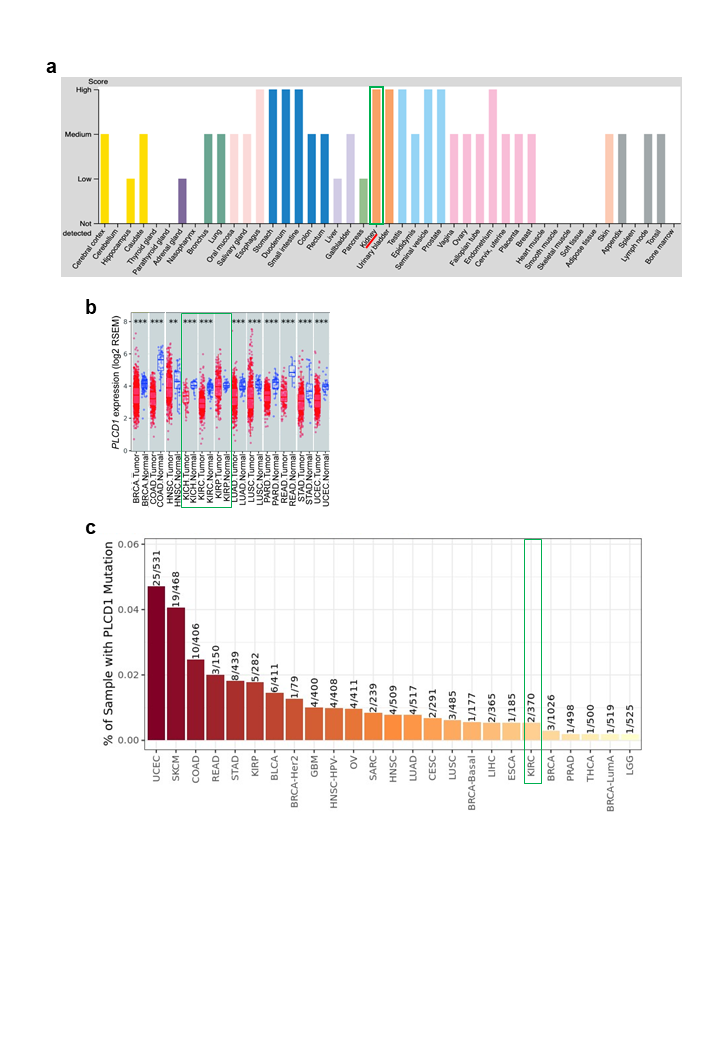

Supplement: Supplementary file 1 — Additional file 1: Overview of PLCD1 bioinformatics analysis. a Protein expression of PLCD1 in 45 kinds of tissues through Human Protein Atlas. b Differential expression of PLCD1 between tumor and adjacent tissues across TCGA tumors in TIMER2.0 database. c PLCD1 mutation rates across TCGA tumors analyzed by TIMER 2.0 database. [file 13148_2023_1448_MOESM1_ESM.tif]
